# Burnout and Metabolic Syndrome in Female Nurses: An Observational Study

**DOI:** 10.3390/ijerph16111993

**Published:** 2019-06-05

**Authors:** Gabriela Chico-Barba, Karime Jiménez-Limas, Bernarda Sánchez-Jiménez, Reyna Sámano, Ana Lilia Rodríguez-Ventura, Rafael Castillo-Pérez, Maricruz Tolentino

**Affiliations:** 1 Departmento de Nutrición y Bioprogramación, Instituto Nacional de Perinatología, Ciudad de México 11000, Mexico; gabyc3@gmail.com (G.C.-B.); ssmr0119@yahoo.com.mx (R.S.); rovalilia@hotmail.com (A.L.R.-V.); armando.castillo1958@gmail.com (R.C.-P.); cruz_tolentino@yahoo.com.mx (M.T.); 2 Escuela de Enfermería, Facultad de Ciencias de la Salud, Universidad Panamericana, Ciudad de México 03920, Mexico; 3 Subdirección de Investigación en Intervenciones Comunitarias, Instituto Nacional de Perinatología, Ciudad de México 11000, Mexico; emiberna20@yahoo.com.mx

**Keywords:** nurses, burnout, metabolic syndrome, waist circumference, emotional exhaustion, personal accomplishment, Mexico

## Abstract

Nurses are at risk of having burnout due to workload and job stress—studies have reported that chronic stress is associated with metabolic syndrome. This study aimed to assess the association between burnout and metabolic syndrome in a sample of female nurses. Data were collected from a cross-sectional study from 2016 to 2018 in a tertiary hospital in Mexico City. All nurses that work in the hospital were invited to participate. Information pertaining to sociodemographic (age, education level), work (labor seniority, service area, shift work), anthropometric (weight, waist circumference, blood pressure) and biochemical (glucose, serum lipids) variables were collected. Burnout was assessed using the Maslach Burnout Inventory test, and metabolic syndrome was defined according to the International Diabetes Federation criteria. A total of 168 nurses participated with a median age of 44 years. The prevalence of burnout and metabolic syndrome was 19.6% and 38.7%, respectively. There was no association between burnout and metabolic syndrome (*p* = 0.373). However, associations of emotional exhaustion (aOR: 14.95; 95% CI: 1.5–148.7), personal accomplishment (aOR: 0.13; 95% CI: 0.01–0.99), and night shift (aOR: 12.39; 95% CI: 1.02–150.5) with increased waist circumference were found. Strategies are needed to prevent burnout and metabolic syndrome in nurses, especially in those who work at night shift.

## 1. Introduction

Burnout is a syndrome of emotional exhaustion, depersonalization, and low personal accomplishment, which is acquired by workers who have direct contact with customers and users [1]. Among the healthcare professionals, nurses are especially at risk of developing burnout due to the high workload and job stress that is mostly caused by working proximity to patients and taking care of them [2].

Some studies have shown that chronic stress is associated with metabolic syndrome in animal models [3] and clinical settings [4,5,6], but little is known about the relationship between burnout and metabolic syndrome.

There is evidence that burnout has an impact not only to the emotional status [7] and self-esteem [8] but also on metabolic profile. Cross-sectional studies have shown an association between cardiovascular risk factors and burnout [9,10]. On the other hand, a longitudinal study found an association between the risk factors for arteriosclerotic disease and the presence of burnout in healthy, middle-aged men [11]—burned out men had significant increases in their waist circumference and body weight at 4–5 years of follow-up.

The possible pathway between burnout and metabolic syndrome could be explained by the hypothalamic–pituitary–adrenal (HPA) axis. The HPA axis remains hyperactive in the presence of burnout due to exposure to chronic stress, resulting in fat accumulation [12,13]. Body fat mass is a risk factor for cardiometabolic diseases [14,15]. 

As health professionals, nurses should have good health to take care of the patients and also to be a health promoter, as they are role models, advocates, and educators. Nurses are exposed to different risk factors for non-chronic diseases, for instance, stress and anxiety as a result of their workload and daily interaction with sick people, low physical activity, and long working hours [16,17,18]. Their job characteristics make it difficult for nurses to have healthy habits, as studies have reported low self-care in nurses [16,17,18]. There is also a high prevalence of metabolic syndrome [19]. For the reasons mentioned above, the aim of this study was to assess the association between burnout and metabolic syndrome in a sample of female nurses.

## 2. Materials and Methods 

### 2.1. Study Design

This observational, cross-sectional study included nurses who work at the Instituto Nacional de Perinatología (National Perinatology Institute, INPer) in Mexico City. Sampling was convenience, non-probabilistic, and based on consecutive cases that met the following inclusion criteria: to be a formal institutional employee, any age, any shift, and area of service. Nursing students, practitioners, and rotating personnel were excluded from the study, as well as pregnant women. Participants were enrolled from 2016 to 2018, with 509 nurses invited to participate, but only 171 (33%) joined the study. As only three men participated, they have been eliminated from the final sample, in addition to participants with incomplete questionnaires. The total sample consisted of 168 female nurses.

### 2.2. Sociodemographic Data and Working Information

The information regarding age, educational level, socioeconomic status, marital status, working shift, the area of service, labor seniority, having children, and having more than one job was obtained through a questionnaire.

### 2.3. Anthropometric Assessment

The method of measurement was standardized and performed by fixed personnel. Weight was obtained using a digital scale (Tanita Terraillon, 100 g of precision) and height was assessed using a stadiometer (SECA 231, 0.1 cm of precision). Body mass index (BMI) was calculated by dividing weight in kilograms by the height in square meters and was categorized according to the World Health Organization cutoff points [20]. Waist circumference was measured using a non-extensible tape (SECA 201, 1 mm of precision); the participants were asked to stand with their arms raised and abdomen uncovered, the tape was set at the midpoint between the last rib and the iliac crest, and after a normal exhalation, the measurement was taken. Blood pressure was obtained using a mercury sphygmomanometer in the non-dominant arm in the seated position after 5 min of resting, according to international standards [21]. Two measurements were made and the average was recorded.

### 2.4. Biochemical Analyses

Blood samples were obtained from 7:00 to 8:00 in the morning, after fasting for 12 h. Serum samples were frozen at −70 °C until the determination of glucose, glycated hemoglobin (HbA1c), total cholesterol, high-density lipoprotein (HDL) cholesterol, and triglycerides. Participants with an HbA1c >5.7% were given an oral glucose tolerance test (OGTT), as this cutoff point is an indicator of increased risk for diabetes, according to the American Diabetes Association guidelines [22]. For the OGTT, an amount of 75 g of glucose was used, and the reading was taken 2h post-test.

### 2.5. Burnout Assessment

Burnout was assessed using the Maslach Burnout Inventory—Human Services Survey test [23]. The questionnaire consists of 22 items, which evaluates three domains: emotional exhaustion (9 items), depersonalization (5 items), and personal accomplishment (8 items). Each item is assessed on a Likert scale, scoring from 0 to 6. A score was calculated by the sum of points of the items of each domain. Emotional exhaustion and depersonalization have a direct relationship with burnout while, contrarily, personal accomplishment has a negative relation. Even though Maslach proposed to classify each domain as low, mild, or high, it does not give a cutoff point to determine burnout as present or absent. For this study, the total points of each domain were divided into tertiles, and burnout was defined as tertile 3 of emotional exhaustion plus tertile 3 of depersonalization and/or tertile 1 of personal accomplishment, according to Kitaoka-Higashiguchi [11].

### 2.6. Metabolic Syndrome Definition

Metabolic syndrome was defined according to the International Diabetes Federation (IDF) [24]. The criteria are central obesity plus any two of the following: (1) triglycerides ≥150 mg/dL (1.7 mmol/L) or specific treatment for this lipid abnormality; (2) HDL cholesterol <50 mg/dL (1.29 mmol/L) in females or specific treatment for this lipid abnormality; (3) systolic blood pressure ≥130 mmHg or diastolic blood pressure ≥85 mm Hg or treatment of previously diagnosed hypertension; and (4) fasting plasma glucose ≥100 mg/dL or previously diagnosed type 2 diabetes. Central obesity was defined as waist circumference ≥80 cm.

### 2.7. Ethics

The Institutional Review Boards and Ethics Committees from Instituto Nacional de Perinatología (Reg. 212250-3300-11402-01-15) and Facultad de Ciencias de la Salud Universidad Panamericana (Reg. CIEE-001-2017-01) approved the study. Data gathering was confidential, taking into account ethical issues such as autonomy and respect for persons. The guidelines of the Helsinki Declaration were followed. The identification information of the patients was replaced by a folio number, to ensure the confidentiality of the data. All participants received nutritional attention at INPer. If necessary, they also received psychological and medical attention.

### 2.8. Statistical Analysis

We performed a descriptive analysis of the characteristics of the study population. Metabolic syndrome factors were categorized into two categories, according to IDF cutoff points (e.g., triglycerides ≥150 mg/dL), and frequencies and percentages were calculated for each factor. A Chi-square test was used to determine the association between burnout and metabolic syndrome. Logistic regression models were performed using the metabolic syndrome factors separately as dependent variables and burnout domains in tertiles as independent variables. The reference categories were tertile 1 for emotional exhaustion and depersonalization, and tertile 3 of personal accomplishment. The models were adjusted for sociodemographic, anthropometric and working variables. All statistical analyses were carried out using IBM SPSS Statistics for Windows, Version 20.0 (IBM Corp, Armonk, NY, USA). Statistical significance was considered at *p <* 0.05. 

## 3. Results

A total of 168 nurses participated in the study. Table 1 shows that the median age of the participants was 44 years. Most of them were married (61.3%) and had children (74.4%). The most frequent education level was college (37.5%), followed by technician (28.6%). It was found that 60.7% of the nurses worked in intensive care units. More than half of our sample belonged to the day shift (61.9%) and had only one job (89.9%). 

**Figure 1:**
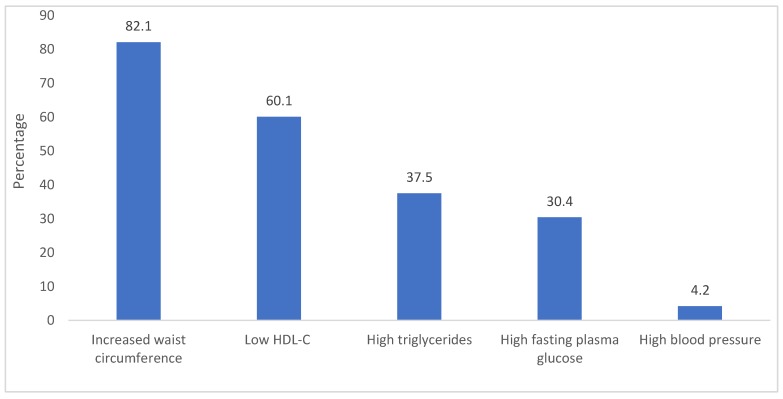
Prevalence of metabolic syndrome criteria in the total sample of nurses. HDL-C: high-density lipoprotein cholesterol.

The prevalence of burnout and metabolic syndrome in the sample were 19.6% and 38.7%, respectively. Regarding metabolic syndrome criteria, 82.1% of the nurses had increased waist circumference (82.1%), followed by low HDL cholesterol (60.1%). Only 4.2% had high blood pressure (Figure 1).

There was no association between burnout and metabolic syndrome (*p* = 0.373). However, an association between the burnout domains and components of the metabolic syndrome factors was found (Table 2). 

Nurses in tertile 2 of emotional exhaustion had a higher risk of having increased waist circumference (adjusted OR: 14.95; 95% CI: 1.5–148.7; *p* = 0.021), compared to tertile 1. Contrarily, nurses in tertile 2 of personal accomplishment had a lower risk of having increased waist circumference (aOR: 0.13; 95% CI: 0.01–0.99; *p* = 0.049), compared to tertile 1. Also, nurses who work in the night shift had a higher risk of having increased waist circumference (aOR: 12.39; 95% CI: 1.02–150.5; *p* = 0.048). The service area did not show an association with increased waist circumference. There was no association between the burnout domains and the other metabolic syndrome criteria (Table 2).

## 4. Discussion

In this sample of Mexican female nurses, the prevalence of burnout (19.6%) was lower than that reported in other studies. Miranda-Lara et al. found a 33.8% rate of burnout in a similar sample of Mexican nurses [25]. We have considered different possible explanations for this difference. The first one is that the Maslach Burnout Inventory classifies the level to which the syndrome domains are found (low, mild, or high) but it does not give a cutoff point to determine burnout [23]. The second explanation is that the study performed by Miranda-Lara et al. does not mention the cutoff points used to define the presence of burnout, so the figures cannot be comparable. Finally, more than half of our sample (66%) had a high education level—it has been demonstrated in other studies that women with high burnout scores were more likely to have lower education levels [26]. 

Regarding the metabolic syndrome, the prevalence in our study was lower than the reported for Mexican female adults [27] (38.7% vs. 52.7%), but similar when compared to nurses from other countries. In a sample of Brazilian nurses, the prevalence of metabolic syndrome was 38.1% [9]. Again, we consider the presence of a high education level as a possible explanation, as it has been reported that the lower the education, the higher the frequencies of metabolic syndrome [27,28]. The reason may be because a low education level could cause differences in opportunities for workers to access health services and affect health-related behaviors. In particular, the lower the socioeconomic status of female workers (which usually may depend on education level), the less they care about healthcare and, hence, the higher the risk of metabolic syndrome [28].

When analyzing the metabolic syndrome criteria separately, we found that the prevalence of increased waist circumference in our study (82.1%) was close to the 87.8% reported for Mexican adult women, described in the 2016 Halfway National Health and Nutrition Survey (Ensanut MC 2016) [29]. Moreover, waist circumference was higher in those who worked night shifts. Other studies report similar findings, as working at night is associated with an increased risk of obesity [30], cardiovascular diseases [31], and cancer [32]. 

On the other hand, the low prevalence of hypertension found in this study (4%) was surprisingly low compared to the 25.5% [29] and 32% reported for the Mexican population and Brazilian nurses, respectively [33]. The reasons behind this low prevalence are still being studied. We consider that the nurses that already had hypertension did not want to participate in the study because they wanted their health information to remain confidential. We came to this assumption after having informal and confidential interviews with some nurses after their participation in the study—they said that they prefer to hide their hypertension diagnosis. There is little evidence regarding this issue. The World Medical Association, in their statement on physician well-being, says that physicians hide their diseases because of denial, confidentiality issues, aversion to the patient role, fear of disciplinary action, and loss of performance-based payment, among others [34]. We believe that the same phenomenon occurs with nurses, as they act as role models in healthcare.

As for the association between burnout domains and metabolic syndrome criteria separately, we found that low emotional exhaustion was associated with increased waist circumference. There is no study that has ever tried to associate these variables. Other articles have related the burnout syndrome with an increase in abdominal adiposity [35], waist circumference, body weight, and BMI [11], but no one has ever reported associations with a specific dimension of burnout. An explanation for this finding is that emotional exhaustion is associated with stress [36,37], and stress increases the risk of metabolic syndrome [4]. 

Regarding the association of mild personal accomplishment and lower odds of having high waist circumference, other studies reported similar findings by showing that low personal accomplishment was associated with sedentarism in adults [38]. Sedentarism may lead to increased waist circumference and overweight. Another study found that low self-esteem was associated with unhealthy lifestyle (less of 3 days per week and/or less of 30 min per session of physical activity and poor dietary habits) and, therefore, with increased adiposity [39]. 

Strategies are needed to prevent burnout and metabolic syndrome, and an exercise program may improve both. A previous interventional study explored the impact of an exercise program in banking and insurance workers. The study showed that the workers in the high-intensity exercise program had decreased burnout indicators and their systolic blood pressure was reduced, compared to the control group. In addition, systolic blood pressure was independently associated with burnout and exercise intensity in the crude model, but this was not significant in the adjusted model [40]. Nevertheless, strategies like this should be adapted to nurses’ job and lifestyle, especially in those who work at night shift. Another intervention that has shown beneficial effects in health is yoga, with evidence from randomized control trials and systematic reviews showing that yoga reduced burnout, specifically emotional exhaustion and depersonalization [41,42], and improved cardiometabolic parameters, like fasting blood glucose, triglycerides, and blood pressure [43,44,45].

Regarding the pathway between burnout and metabolic syndrome, future studies should adequately assess biomarkers involved in the activation of the HPA axis, like serum cortisol. Even though cortisol levels increase when the HPA axis is activated in chronic stress [12], there is inconsistent evidence about the levels of cortisol and burnout. The evidence from different systematic reviews and meta-analysis showed that the comparability of the studies is limited due to poor quality assessments of cortisol and burnout [46,47].

One of the strengths of this study is that, to our knowledge, it is one of the few studies that assess the association of burnout and metabolic syndrome. Also, for the assessment of burnout, we used the Maslach Burnout Inventory, which continues to be the most widely used instrument and is considered to be the gold standard. Moreover, we had a high percentage of participation (33%) from nurses, since a 15% participation has been reported in other studies [40]. However, it is likely that non-participants may have had pre-existing cardiovascular diseases and, thus, did not wish to participate in the study. Another possible reason for not participating is that often the number of nurses in each service is inadequate, so they cannot leave their service to participate in our study assessments.

The sample size is one of the limitations of our study, as it is likely that a greater sample size would result in narrower confidence intervals and burnout domains to be associated with other metabolic syndrome criteria. Also, our sample is not representative of all Mexican nurses as it consists only of female nurses of a tertiary hospital. Regarding the cross-sectional design, we cannot assume causality in the associations that we found.

## 5. Conclusions

Amongst female nurses working in a tertiary hospital, there was no association between burnout and metabolic syndrome. Nevertheless, nurses in tertile 2 of emotional exhaustion had a higher risk of having increased waist circumference, and nurses in tertile 2 of personal accomplishment had a lower risk of having increased waist circumference. Strategies are needed to prevent burnout and metabolic syndrome in female nurses, especially in those who work at night shift.

## Figures and Tables

**Table 1:** General characteristics of the total sample of nurses.

Category	Variable	n (%)
Sociodemographics	Age (years) ^1^	44 (38–50)
	Marital status	
	Single	65 (39)
	Married	103 (61)
	Educational level	
	Technician	57 (34)
	College	63 (38)
	Graduate	48 (28)
	Having children	
	Yes	125 (74)
	No	43 (26)
Working related variables	Labor senority (years) ^2^	24 (16–28)
	Working years in current service	4 (2–13)
	Service area	
	Intensive care units	102 (61)
	Inpatients	20 (12)
	Outpatients	22 (13)
	No contact with patients	24 (14)
	Shift work	
	Day (8-hour length)	104 (62)
	Mid-day (7-hour length)	14 (8)
	Night(12-hour length)	50 (30)
	More than one job	
	Yes	17 (10)
	No	151 (90)

^1^ Median (P25–P75). ^2^ Service area according to the type of patients attended.

**Table 2:** Association of burnout domains with metabolic syndrome factors.

Independent Variables	Increased Waist Circumference			Low HDL-C			High Triglycerides			High Fasting Plasma Glucose			High Blood Pressure		
	aOR	95% CI	*p*	aOR	95% CI	*p*	aOR	95% CI	*p*	aOR	95% CI	*p*	aOR	95% CI	*p*
Emotional exhaustion															
T1	Reference			Reference			Reference			Reference			Reference		
T2	**14.95**	**1.50–148.71**	**0.021**	0.84	0.35–2.01	0.710	1.26	0.53–3.01	0.593	0.54	0.21–1.38	0.199	0.43	0.03–5.33	0.513
T3	3.57	0.70–18.15	0.125	0.66	0.28–1.57	0.356	1.24	0.52–2.95	0.613	0.40	0.15–1.06	0.067	2.35	0.25–21.7	0.450
Depersonalization															
T1	Reference			Reference			Reference			Reference			Reference		
T2	4.28	0.62–29.65	0.140	1.45	0.63–3.35	0.380	1.17	0.51–2.65	0.706	1.11	0.44–2.76	0.816	0.42	0.05–3.52	0.425
T3	1.60	0.25–10.24	0.615	1.10	0.47–2.54	0.822	0.91	0.39–2.14	0.838	1.60	0.63–4.04	0.316	0.23	0.01–3.80	0.308
Personal accomplishment															
T1	0.26	0.03–2.36	0.234	1.20	0.50–2.84	0.673	0.61	0.25–1.46	0.271	0.80	0.30–2.08	0.653	0	0–0	0.997
T2	**0.13**	**0.01–0.99**	**0.049**	0.77	0.33–1.80	0.551	0.77	0.33–1.78	0.546	2.10	0.86–5.15	0.103	1.60	0.25–9.98	0.611
T3	Reference			Reference			Reference			Reference			Reference		
Shift															
Day	Reference			Reference			Reference			Reference			Reference		
Mid-day	13.93	0.52–369.9	0.115	2.58	0.59–11.2	0.206	0.61	0.13–2.84	0.534	1.19	0.28–5.09	0.810	0.82	0.3–18.71	0.904
Night	**12.39**	**1.02–150.5**	**0.048**	0.55	0.24–1.25	0.156	1.08	0.47–2.46	0.846	0.97	0.39–2.38	0.948	0.75	0.08–6.97	0.805

aOR: adjusted odds ratio, CI: confidence interval, HDL-c: high density lipoprotein cholesterol. Logistic regression models adjusted by age, service area, and body mass index. Bold numbers show statistically significant associations.
